# Carbon ion radiotherapy and radiation-induced lung injury: clinical evidence, mechanistic insights, and future directions

**DOI:** 10.3389/fonc.2026.1833538

**Published:** 2026-05-29

**Authors:** Yiqin Qiu, Guoqiang Zhong, Yongzhen Li, Wei Feng

**Affiliations:** 1 Postgraduate Training Base Alliance of Wenzhou Medical University (Zhejiang Cancer Hospital), Hangzhou, China; 2 College of Clinical Medicine, Hebei University of Engineering, Handan, China; 3 Hebei University of Engineering School of Clinical Medicine, Handan, China; 4 Department of Thoracic Radiotherapy, Zhejiang Cancer Hospital, Hangzhou, China; 5 Hangzhou Institute of Medicine (HIM), Chinese Academy of Sciences, Hangzhou, China

**Keywords:** carbon ion radiotherapy, high linear energy transfer, lung cancer, radiation-induced lung injury, radiobiological mechanisms

## Abstract

Carbon ion radiotherapy (CIRT), owing to its unique physical dose distribution and high linear energy transfer (high-LET) characteristics, has shown remarkable advantages in the treatment of thoracic tumors in recent years. Compared with photon and proton radiotherapy, CIRT not only offers a higher relative biological effectiveness, enabling efficient eradication of hypoxic and radioresistant tumor cells, but also significantly reduces the radiation dose to surrounding normal lung tissue while maintaining excellent local control, thereby lowering the risk of radiation-induced lung injury (RILI). Clinical evidence indicates that CIRT demonstrates favorable tolerability and a lower incidence of radiation pneumonitis across various patient groups. Mechanistic evidence further suggests that the reduced risk of RILI with CIRT may arise from a combination of factors, including superior dose distribution and distinct high-LET radiobiological effects that induce complex DNA damage in tumor cells. In addition, carbon ion irradiation may influence key biological processes involved in lung injury, such as oxidative stress responses, inflammatory signaling, and profibrotic pathways, including TGF-β activation. Although current findings are largely derived from limited clinical cases and preclinical studies, they collectively highlight the potential of CIRT as an optimal approach to achieve effective tumor control while mitigating RILI. Future multicenter prospective studies and international collaborations are urgently needed to further clarify the protective effects and clinical value of CIRT, together with technological advances in beam delivery and adaptive treatment planning, as well as continued exploration of its integration with immunotherapy.

## 1 Introduction

Radiotherapy is a cornerstone in managing malignant tumors, especially thoracic malignancies like lung cancer, where it enhances local tumor control and patient survival (1, 2). However, photon-based radiotherapy (e.g., X-rays) is limited by its physical properties, which inevitably expose surrounding normal tissues to low- to medium-dose radiation. This diffuse exposure causes acute and chronic toxicities, with radiation-induced lung injury (RILI)—including radiation pneumonitis (RP) and pulmonary fibrosis—being among the most common and severe (3–5). These complications diminish quality of life and can be life-threatening.

Efforts to address these limitations have led to advanced modalities like particle therapy, with carbon ion radiotherapy (CIRT) emerging as a promising heavy-ion option due to its superior physical and biological properties. Yet, clinical experience with CIRT is limited compared to photon therapy, relying mostly on small, single-center studies. Thus, the long-term efficacy and safety of CIRT warrant deeper investigation.

This review synthesizes evidence on CIRT’s potential to reduce RILI. We explore how its distinctive dose distribution and biological effects offer lung protection at multiple levels, from macroscopic delivery to cellular and molecular regulation. Key topics include precise dose deposition, oxidative stress control, pro-fibrotic pathway suppression. We also evaluate clinical studies on CIRT’s use in various lung cancer stages and high-risk groups, while highlighting challenges and future directions. By integrating these insights, we offer radiation oncologists, radiobiologists, and researchers an updated view of CIRT’s protective role, promoting its use to optimize tumor control and minimize toxicity.

## 2 Physical and biological characteristics of CIRT

### 2.1 Physical dose deposition and normal lung sparing

As is well recognized, lung tissue is composed of a large population of alveolar epithelial cells and vascular endothelial cells, both of which are highly susceptible to radiation-induced DNA damage and apoptosis. Compared with conventional photon radiotherapy, carbon ion irradiation may offer potential advantages in addressing these vulnerabilities (6). Unlike photon radiotherapy, which exhibits exponential attenuation along its path, carbon ions deposit relatively low energy in the entrance region, followed by a sharp increase near the end of their range, forming the *Bragg peak* (7). Beyond this peak, the primary carbon ion dose falls off rapidly; however, nuclear fragmentation generates lighter secondary particles that extend beyond the nominal range, producing a distal fragmentation tail. In addition, due to their relatively large mass, carbon ions experience less lateral scattering than photons or protons, resulting in a more confined dose distribution (8). This physical property enables the delivery of conformal dose distributions that can be adapted to tumor geometry, thereby reducing the volume of normal lung exposed to low- or intermediate-dose radiation, a key dosimetric factor associated with the risk of RP (9).

In addition, several studies have confirmed the dosimetric benefits of CIRT (9–12). Shirai et al. reported that, compared with three-dimensional conformal radiotherapy (3D-CRT) and intensity-modulated radiotherapy (IMRT), CIRT significantly reduced key lung dose–volume parameters such as V_5_, V_10_, and V_20_. This reduction in low- and intermediate-dose exposure is particularly important for lung tissue, as it lowers the likelihood of subclinical injury that could accumulate over time. Another comparative study demonstrated that, relative to volumetric modulated arc therapy (VMAT), CIRT provided superior sparing of normal tissues, with quantifiable improvements in metrics like mean lung dose (MLD) and the percentage of lung receiving doses above 10 Gy. These dosimetric advantages are expected to translate into clinical benefits, such as a potential reduction in the risk of RP, by limiting the radiation exposure to non-target lung regions.

### 2.2 Biological characteristics

Carbon ions are a form of high linear energy transfer (high-LET) radiation, characterized by substantially greater biological effectiveness compared with conventional photon irradiation. This enhanced potency arises from their ability to induce dense ionization tracks and complex DNA damage, including clustered lesions and a higher incidence of double-strand breaks, which are less amenable to cellular repair. As a result, carbon ions can achieve more efficient tumor cell killing at equivalent physical doses (13). Supporting this concept, Liu et al. (14) reported a TD_50_ of 15.70 Gy for grade ≥2 acute RP following carbon-ion radiotherapy, whereas the TD_50_ for photon radiotherapy was 41.79 Gy, corresponding to an estimated RBE of 2.66 for lung toxicity.

A key feature of carbon ion irradiation is its elevated relative biological effectiveness (RBE), typically estimated to be two- to three-fold higher than that of photons or protons. In addition, high-LET radiation exhibits reduced dependence on oxygenation status and cell-cycle phase, making it particularly advantageous for targeting hypoxic and radioresistant tumor subpopulations (15).

These biological characteristics provide a strong rationale for the use of CIRT in thoracic malignancies, where tumor hypoxia and intrinsic radioresistance often limit the effectiveness of conventional radiotherapy. However, it should be noted that the biological effectiveness of carbon ions is not fixed but varies with multiple physical and biological factors, including linear energy transfer (LET), dose, and tissue characteristics. In clinical practice, treatment planning is therefore based on RBE-weighted dose derived from radiobiological models integrated into treatment planning systems. Notably, RBE varies spatially within the irradiation field, particularly across the spread-out Bragg peak, rather than being uniform (16). As a result, the accurate representation of RBE in clinical settings remains subject to modeling assumptions and limited tissue-specific data. These uncertainties, particularly in normal lung tissue, will be discussed in detail in Section 6.1.1.

## 3 Pathogenesis and evaluation of RILI

### 3.1 Overview of RILI

#### 3.1.1 Definition and stages

RILI is a prevalent late complication of thoracic radiotherapy, particularly in patients treated for lung, breast, or esophageal cancers. It can severely diminish the quality of life and restrict dose escalation, potentially undermining treatment efficacy. RILI unfolds as a dynamic pathological process, typically categorized into two main stages: acute RP and chronic radiation-induced pulmonary fibrosis (RPF).

The acute phase typically occurs within 1–6 months after radiotherapy (17, 18). Pathologically, it involves damage to alveolar epithelial cells and capillary endothelial cells, triggering localized inflammation. Clinically, symptoms may include cough, dyspnea, chest tightness, and low-grade fever, which can escalate to hypoxemia in severe cases. After six months, the process shifts to tissue remodeling and fibrosis, marked by fibroblast activation and excessive extracellular matrix (ECM) deposition. Patients often experience worsening dyspnea, with radiological signs such as linear opacities, bronchial traction, and honeycombing (19).

#### 3.1.2 Mechanisms of RILI

The pathogenesis of RILI is multifaceted, encompassing cellular injury, immune responses, inflammatory cascades, and oxidative stress. It can be broadly conceptualized as three interconnected processes: radiation-induced cellular damage, inflammatory activation, and fibrotic progression. Understanding these mechanisms in detail is crucial for identifying therapeutic targets and mitigating injury.

##### 3.1.2.1 Radiation-induced cellular injury

Ionizing radiation directly causes DNA damage in alveolar epithelial cells and vascular endothelial cells, including base loss, single-strand breaks (SSBs), and double-strand breaks (DSBs) (20). These lesions disrupt cellular replication and repair processes, leading to apoptosis (programmed cell death) or necrosis (uncontrolled cell lysis). For instance, DSBs activate DNA repair pathways like non-homologous end joining (NHEJ) or homologous recombination, but persistent damage overwhelms these systems, triggering cell cycle arrest or death. Concurrently, radiation induces water radiolysis, producing reactive oxygen species (ROS) such as superoxide anions (O_2_^-^), hydrogen peroxide (H_2_O_2_), and hydroxyl radicals. These free radicals further damage lipids (e.g., peroxidation of cell membranes), proteins (e.g., enzyme inactivation), and nucleic acids (e.g., further DNA adduct formation), amplifying oxidative stress and tissue injury (21, 22). This oxidative burst also impairs mitochondrial function, releasing additional ROS in a vicious cycle that exacerbates endothelial permeability and alveolar barrier disruption, contributing to edema and inflammation.

##### 3.1.2.2 Immune and inflammatory cascades

Damaged cells release damage-associated molecular patterns (DAMPs), such as high-mobility group box 1 (HMGB1) protein, ATP, and mitochondrial DNA, which act as alarm signals to recruit inflammatory cells such as neutrophils, monocytes, and macrophages into lung tissue. Neutrophils are among the first responders, migrating via chemotactic gradients to phagocytose debris and release proteases that further degrade tissue. Monocytes differentiate into macrophages, while lymphocytes (e.g., T cells) infiltrate, amplifying the response. These infiltrating cells secrete proinflammatory cytokines, including tumor necrosis factor-α (TNF-α), interleukin-1 (IL-1), interleukin-6 (IL-6), interleukin-13 (IL-13), and interferon-γ (IFN-γ), thereby propagating immune cascades (23). The NF-κB signaling pathway plays a central role in this phase, as radiation activates it through ROS-mediated inhibition of IκB (the inhibitor of NF-κB), leading to nuclear translocation of NF-κB dimers and upregulation of cytokine genes. This creates a self-sustaining inflammatory milieu, where cytokines recruit more immune cells, forming a feedback loop that drives acute RP (24).

##### 3.1.2.3 Initiation and persistence of fibrosis

As inflammation transitions to the chronic phase, transforming growth factor-β (TGF-β), a key profibrotic cytokine, is persistently activated via pathways like Smad signaling, promoting fibroblast activation and proliferation. TGF-β induces myofibroblast differentiation (characterized by α-smooth muscle actin expression), enhances collagen synthesis (e.g., types I and III), and stimulates ECM deposition while suppressing matrix metalloproteinases that degrade ECM, ultimately driving fibrogenesis (25–28). This leads to chronic fibrosis, where fibroblasts and myofibroblasts accumulate in the lung interstitium, producing collagen and ECM that disrupt alveolar architecture and impair gas exchange.

##### 3.1.2.4 Macrophage polarization

Early stages are dominated by M1 macrophage-mediated inflammatory responses (pro-inflammatory, producing IL-12 and TNF-α), whereas later stages shift toward M2 macrophage-driven profibrotic effects (anti-inflammatory, secreting IL-10 and arginase), promoting fibrosis through TGF-β amplification (29). An imbalance between Th1 (pro-inflammatory, IFN-γ-producing) and Th2 (anti-inflammatory, IL-4/IL-13-producing) T-cell subsets further exacerbates this, with Th2 cytokines like interleukin-4 (IL-4) and IL-13 enhancing TGF-β signaling and myofibroblast transdifferentiation (30). Conversely, endogenous antifibrotic mediators, such as prostaglandin E_2_ (PGE_2_), may counteract fibrosis by binding to EP2/EP4 receptors on fibroblasts, inhibiting TGF-β expression and reducing collagen production in certain contexts (31).

**Figure 1:**
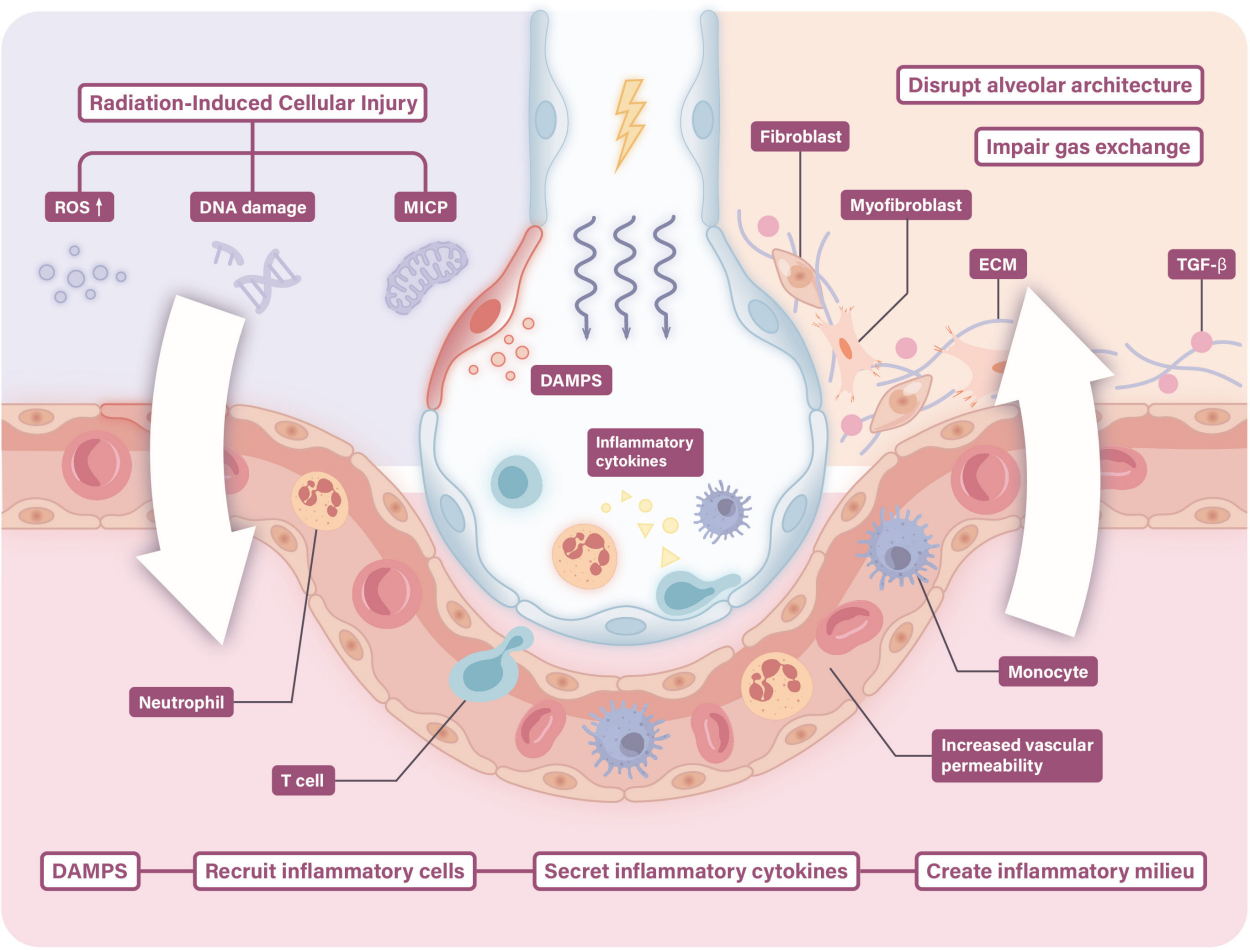
Pathogenesis of RILI. RILI develops through three interconnected stages. First, ionizing radiation induces DNA damage in alveolar epithelial and vascular endothelial cells and generates ROS via water radiolysis, leading to oxidative stress, mitochondrial dysfunction, and disruption of the alveolar–capillary barrier. Second, damaged cells release DAMPs, which recruit inflammatory cells and trigger cytokine-mediated immune cascades, ultimately contributing to acute radiation pneumonitis. Third, persistent inflammation activates profibrotic signaling pathways, particularly TGF-β, promoting fibroblast activation, myofibroblast differentiation, and extracellular matrix deposition—processes involved in the pathogenesis of progressive pulmonary fibrosis. DAMPs, damage-associated molecular patterns; RILI, radiation-induced lung injury; ROS, reactive oxygen species; TGF-β, transforming growth factor-β.

Overall, RILI stems from radiation-triggered cellular injury and ROS buildup, which progressively ignite inflammatory networks and fibrotic remodeling. A deeper understanding of these mechanisms is critical for the development of early interventions and targeted therapies to mitigate pulmonary toxicity and improve the safety and efficacy of thoracic radiotherapy. (**Figure 1**).

### 3.2 Association between conventional radiotherapy and RILI

Photon radiotherapy is a mainstay for thoracic malignancies, offering effective tumor control but inherently exposing significant volumes of normal lung tissue to radiation, thereby increasing RILI risk and threatening long-term outcomes. Clinically, RILI incidence after conventional photon therapy varies widely (5–58%), as it is influenced by dose, irradiated volume, fractionation, and patient characteristics (32–34). In conventional 3D-CRT, key dosimetric predictors include the lung volume receiving ≥20 Gy (V20), with elevated RP risk when V_20_ exceeds 35%. Low-dose metrics (e.g., V5, V10) and MLD are also prognostic, with MLD independently linked to ≥grade 3 RP (odds ratio = 1.002, p = 0.03). Notably, there is no universal consensus regarding which dose–volume parameters are most predictive. Reported threshold values for dose–volume histogram (DVH) metrics such as V10, V20, and V30 vary considerably across studies. Therefore, the overall shape of the DVH was selected to provide more comprehensive information for predicting RP risk (4, 35).

Beyond dose-related parameters, treatment modality also plays a role. Concurrent chemoradiotherapy markedly exacerbates pulmonary toxicity, and prior thoracic irradiation increases susceptibility to immune checkpoint inhibitor-related pneumonitis, as seen in higher pulmonary adverse events with PD-1 inhibitors post-radiotherapy (36). Likewise, elderly patients receiving concurrent platinum- or taxane-based chemotherapy are at particularly high risk of RILI (4).

Compared to photons, particle therapy offers dosimetric advantages through the Bragg peak, enabling improved dose conformity to the tumor while sparing surrounding normal tissues. This property is particularly relevant in thoracic radiotherapy, where minimizing normal lung irradiation is critical for reducing the risk of radiation-induced lung injury.

Nevertheless, as with all particle therapies, its application in the thoracic region remains challenged by respiratory motion and associated range uncertainties, which may affect dose accuracy (37, 38). To address these issues, motion management and robustness-enhancing strategies, including four-dimensional imaging, respiratory gating, rescanning, and adaptive planning, have been actively investigated (39–41).

## 4 Clinical outcomes after carbon ion therapy for lung cancer

To provide an overview of clinical outcomes following CIRT for lung cancer, and to place these findings in the context of photon and proton therapies, we summarized published studies reporting pulmonary toxicity and treatment efficacy. Clinical studies published between 2005 and 2025 were identified through searches of Google Scholar and PubMed. The inclusion criteria comprised retrospective studies involving patients with lung cancer that reported clinical outcomes such as local control (LC), overall survival (OS), V20, and the incidence of RP.

### 4.1 Clinical evidence

**Figure 2:**
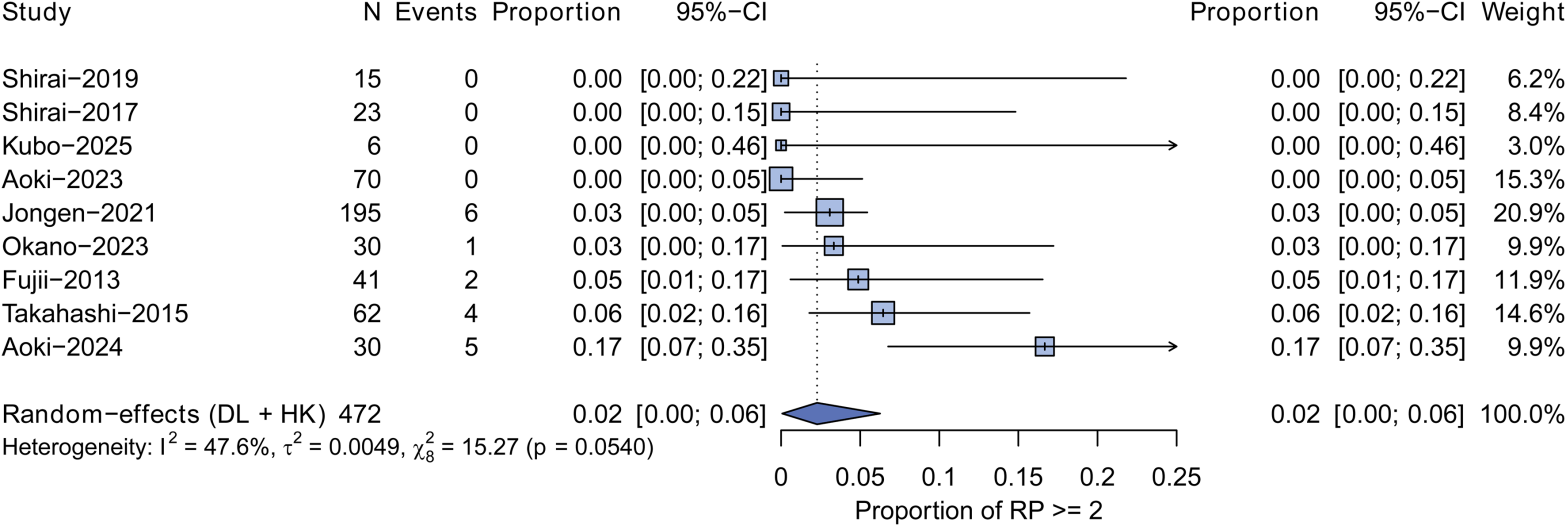
Meta-analysis of the incidence of grade ≥2 RP following carbon ion radiotherapy. The x-axis represents the proportion of grade ≥2 RP; N denotes the total number of patients per study; Events indicates the number of reported grade ≥2 RP events per study. The analysis used a random-effects model (DL and HK methods). The overall pooled incidence of grade ≥2 RP is 0.02 (95% CI: 0.00–0.06). RP, radiation pneumonitis; DL, DerSimonian-Laird; HK, Hartung-Knapp; CI, confidence interval.

A pooled analysis of nine studies including 472 patients estimated an overall incidence of grade ≥2 RP of 2% (95% confidence interval: 0–6%), with moderate heterogeneity (I² = 47.6%, p = 0.054) (**Figure 2**). However, the precision of this estimate is limited by inter-study heterogeneity, small event numbers, and variability in follow-up duration and toxicity assessment. Approximately 67% of the included studies reported no or very few grade ≥2 RP events.

In locally advanced non-small cell lung cancer (NSCLC), available CIRT series have reported rates of severe acute or late pulmonary and esophageal toxicities generally ≤10%. Although these rates are numerically lower than those described in many historical photon- or proton-based studies, cross-modality comparisons should be interpreted cautiously due to substantial differences in patient selection, treatment planning, dose fractionation, and toxicity reporting standards (10, 12, 42, 43).

Favorable tolerability of CIRT has also been reported in selected high-risk clinical scenarios, such as recurrent disease and re-irradiation settings. For instance, a hypofractionated CIRT study in patients with isolated lymph node metastases observed no grade ≥2 RP events and achieved a 2-year LC rate of 92%. In unresectable esophageal cancer, sequential proton plus carbon ion therapy was associated with grade ≥2 and grade ≥3 RP rates of 15% and 5%, respectively. While these findings are encouraging, the lack of prospective comparative data limits definitive conclusions regarding relative toxicity reduction (44).

Patients with lung cancer complicated by interstitial pneumonia (IP) represent a population at particularly high risk for RP. In a retrospective single-fraction CIRT study including 50 patients with stage IA–IIB disease, the incidence of grade ≥2 RP was 12%, with two suspected grade 5 RP events. These findings suggest a potentially acceptable toxicity profile in this high-risk cohort when strict dose constraints are applied. Notably, lung V20 >5% emerged as an independent predictor of OS, underscoring the importance of meticulous treatment planning in patients with IP (45).

Considered together, current evidence suggests that CIRT may be associated with a relatively low incidence of pulmonary toxicity across diverse clinical contexts, including selected high-risk populations. However, the available data are largely retrospective and heterogeneous, and further prospective studies are required to define the true magnitude of any toxicity reduction and to establish optimal patient selection and dose–volume constraints.

### 4.2 Dosimetric advantages in lung tissue sparing

Through analysis of the dosimetric and clinical outcomes reported in the summarized studies, the pulmonary protective characteristics of CIRT can be better understood. In early-stage (Stage I) NSCLC cohorts treated predominantly with radiotherapy alone, lung V20 was generally maintained within 5–8% (e.g., Miyamoto 2007; Shirai 2017/2019), and grade ≥3 RP was infrequently observed. These studies also reported favorable LC, Two- to five-year LC were generally above 80%, with several series reporting values exceeding 90% (11, 46, 47). However, these results should be interpreted in the context of the smaller target volumes and better baseline pulmonary function characteristic of early-stage populations. In the Stage III series (e.g., Kubo 2025), lung V20 increased to approximately 15%, reflecting larger irradiation volumes. Nevertheless, the reported incidence of severe RP in these cohorts remained relatively low (48), suggesting that dose escalation or treatment of larger target volumes did not inevitably translate into excessive pulmonary toxicity.

Compared with photon radiotherapy, CIRT demonstrates a tendency toward lower lung V20 values. In the summarized table, photon-based studies reported V20 ranging from approximately 9% to 35%, particularly in locally advanced disease. Correspondingly, the incidence of grade ≥2 RP in the photon cohorts was generally higher than that reported in most CIRT series. Although differences in patient characteristics and treatment strategies limit direct comparability across studies, the overall trend supports a dosimetric advantage of CIRT over photon techniques. In contrast, comparisons between CIRT and proton therapy do not reveal a clear distinction. Lung V20 values in proton cohorts commonly ranged from 6% to 13%, largely overlapping with those observed in CIRT populations of similar stage. Reported RP rates were variable in both modalities. Based on the currently available data, the evidence supports a broader advantage of particle therapy over photon radiotherapy, but does not demonstrate a definitive pulmonary superiority of CIRT over proton therapy.

It is also noteworthy that reduction in V20 does not invariably prevent RP. In certain populations, particularly those including patients with interstitial lung disease, clinically significant RP was observed despite relatively low V20 values. For example, in lung cancer with interstitial pneumonia (LC-IP) cohorts, moderate RP occurred even when V20 was close to 5% (45). These findings suggest that while lung dose metrics represent a major determinant of pulmonary toxicity, they are not the sole influencing factor. Baseline pulmonary status, target volume, fractionation schedule, and treatment strategy (radiotherapy alone versus combined-modality therapy) may all contribute to the observed variability.

However, substantial heterogeneity exists among studies with respect to patient populations, tumor stage, total dose, fractionation regimens, use of concurrent therapy, and even the V20 calculation. In addition, considerable variation in sample size further affects the stability of reported toxicity rates. Accordingly, the present comparison should be regarded as a descriptive synthesis intended to illustrate the overall trend of CIRT in reducing RILI (**Tables 1**, **2**).

**Table 1:** Carbon ion radiotherapy vs. proton radiotherapy.

Reference	Radiation	Tumor	TD (Gy RBE)	n	Local control, %	OS, %	RA	V20, %	RP (≥G2/≥G3, %)
Shirai, 2017 (11)	C	NSCLC	61.36	23	81 (2y)	70 (2y)	Yes	7.93 ± 3.61	0/0
Hayashi, 2018 (49)	C	NSCLC-LA	72	141	80.3 (2y)	58.7 (2y)	No	—	10.6/3.5
Shirai, 2019 (47)	C	LC	52.8	15	92 (2y)	75 (2y)	Yes	7.30 ± 3.90	0/0
Miyamoto, 2007 (46)	C	Stage I NSCLC	52.8/60.0	79	90 (5y)	45 (5y)	Yes	5.68 ± 2.64	1.3/0
Okano, 2023 (50)	C	NSCLC-ILD	50–69.6	30	88.1 (3y)	48.2 (3y)	Yes	—	3.3/3.3
Kubo, 2025 (48)	C	Stage III NSCLC	64	6	83.3(5y)	—	Yes	15.60	0/0
Aoki, 2023 (51)	C	Stage I NSCLC	40	70	79.9 (2y), 78.2 (3)	84.3 (2y), 72.3 (3y)	Yes	—	0/0
Aoki, 2024 (52)	C	Stage I-II NSCLC	68.4	30	93.1 (1y), 88.7 (3y)	96.7 (1y), 72.4 (3y)	Yes	10.40	16.7/6.7
Aoki, 2024 (45)	C	LC-IP	50	49	77.8 (3y)	45 (3y)	Yes	5.18	12.2/8.2
Fujii, 2013 (53)	C	Stage I NSCLC	52.8–70.2	41	78 (3y)	76 (3y)	Yes	—	4.9/4.9
	P	Stage I NSCLC	52.8–80	70	81 (3y)	72 (3y)	Yes	—	0/0
Iwata, 2010 (54)	C	Stage I NSCLC	52.8	23	82 (3y)	75 (3y)	Yes	7	8.7/0
	P	Stage I NSCLC	80/60	57			Yes	8	12.3/1.8
Makita, 2015 (55)	P	Stage I NSCLC	66/80	32/24	96 (3y)	81.3 (3y)	Yes	6.0 9.5	16.1/1.8
Chang, 2017 (56)	P	Stage I-II NSCLC	87.5	35	85 (3y)	60 (2y), 42.9 (3y)	Yes	—	14.3/2.9
Suh, 2022 (57)	P	Stage I NSCLC	60	93	94 (2y)	83.3 (2y)	Yes	—	7.5/3.2
Bae, 2022 (58)	P	NSCLC	60	34	92.8 (2y)	73.1 (2y)	Yes	7.93 ± 3.61	26.4/17.6
Noh, 2022 (59)	P	NSCLC	64	54	—	71.1 (2y)	No	13.30	—/13
Boyce-Fappiano, 2021 (60)	P	Stage III NSCLC	54	61	83 (5y)	50.9 (5y)	No	16	4.9/3.3
Ning, 2024 (61)	P	NSCLC	66	66	74 (2y)	31 (2y)	No	23	9.0/4.5

C, carbon ion therapy; LC, lung cancer, also stands for lung cancer (Stage III LC); LC-IP, lung cancer with interstitial pneumonia; NSCLC, non-small cell lung cancer; NSCLC-ILD, non-small cell lung cancer associated with interstitial lung disease; NSCLC-LA, locally advanced non-small-cell lung cancer; OS, overall survival; P, proton therapy; RA, radiotherapy alone; RBE, relative biological effectiveness; RP, radiation pneumonitis; TD, total dose; V20, percentage of the total lung volume receiving ≥ 20 Gy of radiation dose, which is a critical dose-volume histogram parameter for predicting radiation pneumonitis. A dash (—) indicates that the data were not reported in the original publication or were not applicable. The number in parentheses (2y, 3y, 5y) denotes the follow-up period in years (y).

**Table 2:** Carbon ion radiotherapy vs. photon radiotherapy.

Reference	Radiation	Tumor	TD (Gy RBE)	n	LC, %	OS, %	RA	V20, %	RP (≥G2/≥G3, %)
Shirai, 2017 (11)	C	NSCLC	61.36	23	81 (2y)	70 (2y)	Yes	7.93 ± 3.61	0/0
Hayashi, 2018 (49)	C	NSCLC-LA	72	141	80.3 (2y)	58.7 (2y)	No	—	10.6/3.5
Shirai, 2019 (47)	C	LC	52.8	15	92 (2y)	75 (2y)	Yes	7.30 ± 3.90	0/0
Miyamoto, 2007 (46)	C	Stage I NSCLC	52.8/60.0	79	90 (5y)	45 (5y)	Yes	5.68 ± 2.64	1.3/0
Okano, 2023 (50)	C	NSCLC-ILD	50–69.6	30	88.1 (3y)	48.2 (3y)	Yes	—	3.3/3.3
Kubo, 2025 (48)	C	Stage III NSCLC	64	6	83.3(5y)	—	Yes	15.60	0/0
Aoki, 2024 (52)	C	Stage I-II NSCLC	68.4	30	93.1 (1y), 88.7 (3y)	96.7 (1y), 72.4 (3y)	Yes	10.40	16.7/6.7
Aoki, 2024 (45)	C	LC-IP	50	49	77.8 (3y)	45 (3y)	Yes	5.18	12.2/8.2
Fujii, 2013 (53)	C	Stage I NSCLC	52.8–70.2	41	78 (3y)	76 (3y)	Yes	—	4.9/4.9
Boyce-Fappiano, 2021 (60)	X	Stage III NSCLC	50	75	78.1 (5y)	37.2 (5y)	No	18	17.3/4.0
Zou, 2020 (62)	X	Stage III LC	61.2	30	44.2 (1y)	—	No	27.98	40.0/13.4
Miyasaka, 2021 (63)	X	NSCLC	48	27	79.1 (3y)	71.6 (3y)	Yes	—	—/3.7
Bae, 2022 (58)	X	Stage I NSCLC	60	168	92.8 (2y)	83.3 (2y)	Yes	8.90 ± 4.45	19.6/11.9
Suh, 2022 (57)	X	Stage III LC	60	93	94.0 (2y)	83.0 (2y)	Yes	—	11.9/1.1
Sue, 2006 (64)	X	NSCLC	63	68	55.3 (1y)	57 (1y)	No	35	—/8
Song, 2024 (65)	X	Stage III NSCLC	50	45	—	100 (1y)	No	20	13.3/2.2
Chang, 2012 (66)	X	Stage I NSCLC	50	130	98.5 (2y)	78.2 (2y)	Yes	—	9.3/2.3

C, carbon ion therapy; TD, total dose; LC, local control, also stands for lung cancer (Stage III LC); LC-IP, lung cancer with interstitial pneumonia; NSCLC, non-small cell lung cancer; NSCLC-ILD, non-small cell lung cancer associated with interstitial lung disease; NSCLC-LA, locally advanced non-small-cell lung cancer; OS, overall survival; RA, radiotherapy alone; RBE, relative biological effectiveness; RP, radiation pneumonitis; V20, percentage of the total lung volume receiving ≥ 20 Gy of radiation dose, which is a critical dose-volume histogram parameter for predicting lung toxicity; X, photon therapy. A dash (—) indicates that the data were not reported in the original publication or were not applicable. The number in parentheses (2y, 3y, 5y) denotes the follow-up period in years (y).

## 5 Current understanding of cellular and molecular mechanisms underlying lung injury in CIRT

### 5.1 TGF-β

TGF-β serves as a pivotal inflammatory mediator in the pathogenesis of both RP and pulmonary fibrosis (67). It not only induces proliferation and differentiation of fibroblasts, promoting synthesis of collagen by fibroblasts, inhibiting synthesis of collagenase and plasminogen activator, and aggregating considerable amounts of inflammatory cells and cytokines, but also facilitates the process of epithelial–mesenchymal transition in tumor tissues. In the context of lung injury, TGF-β’s overactivation can lead to excessive scarring, impairing gas exchange and respiratory function (68).

Animal studies have shown that the temporal expression patterns of TGF-β vary according to the delivered carbon ion dose. While medium- to high-dose carbon ion irradiation (4–6 Gy) was associated with sustained upregulation of TGF-β, low-dose exposure (2 Gy) induced only a transient increase, followed by a marked decline after four months (69). We speculate that the physical dose of carbon ions may influence the progression of persistent fibrotic responses. However, given the variation in LET along the beam path and the non-constant nature of RBE, physical dose alone may not adequately reflect the biological severity of RILI. Ran et al. further supported this hypothesis, demonstrating that in the carbon ion irradiation group, the peak expression of immunosuppressive factors, including IL-10 and TGF-β, occurred at a physical dose of 2 Gy. Notably, this dose-dependent trend was consistent with the RBE value of carbon ion radiation (70). Klinger et al. reported that carbon ion irradiation, characterized by high-LET, induces TGF-β secretion in HUVECs at very low doses, at which no comparable effect was observed following photon radiotherapy, with a plateau in TGF-β induction observed at 2 Gy (71). Moreover, recent study indicates that CIRT can remodel the fibrotic microenvironment by inducing macrophage polarization toward an M1 phenotype, leading to reduced TGF-β production and subsequent inhibition of the TGF-β/SMAD signaling pathway in fibroblasts, ultimately suppressing ECM accumulation while promoting ECM degradation (72). Although this study was conducted in a keloid model, the identified suppression of TGF-β/SMAD signaling and modulation of macrophage polarization are highly relevant to the pathogenesis of RILI, suggesting a potential mechanistic basis by which CIRT may attenuate radiation-induced pulmonary fibrosis.

### 5.2 Reactive oxygen species

Radiation-induced excessive production of ROS may not only facilitate the progression of RILI through biological processes such as inflammation, angiogenesis, programmed cell death, and autophagy, but also activate multiple intracellular signaling pathways and cytokines, thereby promoting the initiation and maintenance of immune and inflammatory responses and driving the development of pulmonary fibrosis (21, 73, 74).

Compared with conventional photon radiotherapy, CIRT demonstrates superior biological advantages in modulating ROS production, spatial distribution, and subsequent oxidative damage. Multiple studies have consistently reported that ROS generation following CIRT is highly localized. High-density ROS clusters are formed along the ion track core, while minimal ROS are produced in the peripheral regions. This pronounced spatial localization of ROS suggests a potential mechanism by which CIRT may limit oxidative damage to surrounding normal tissues (75).

**Figure 3:**
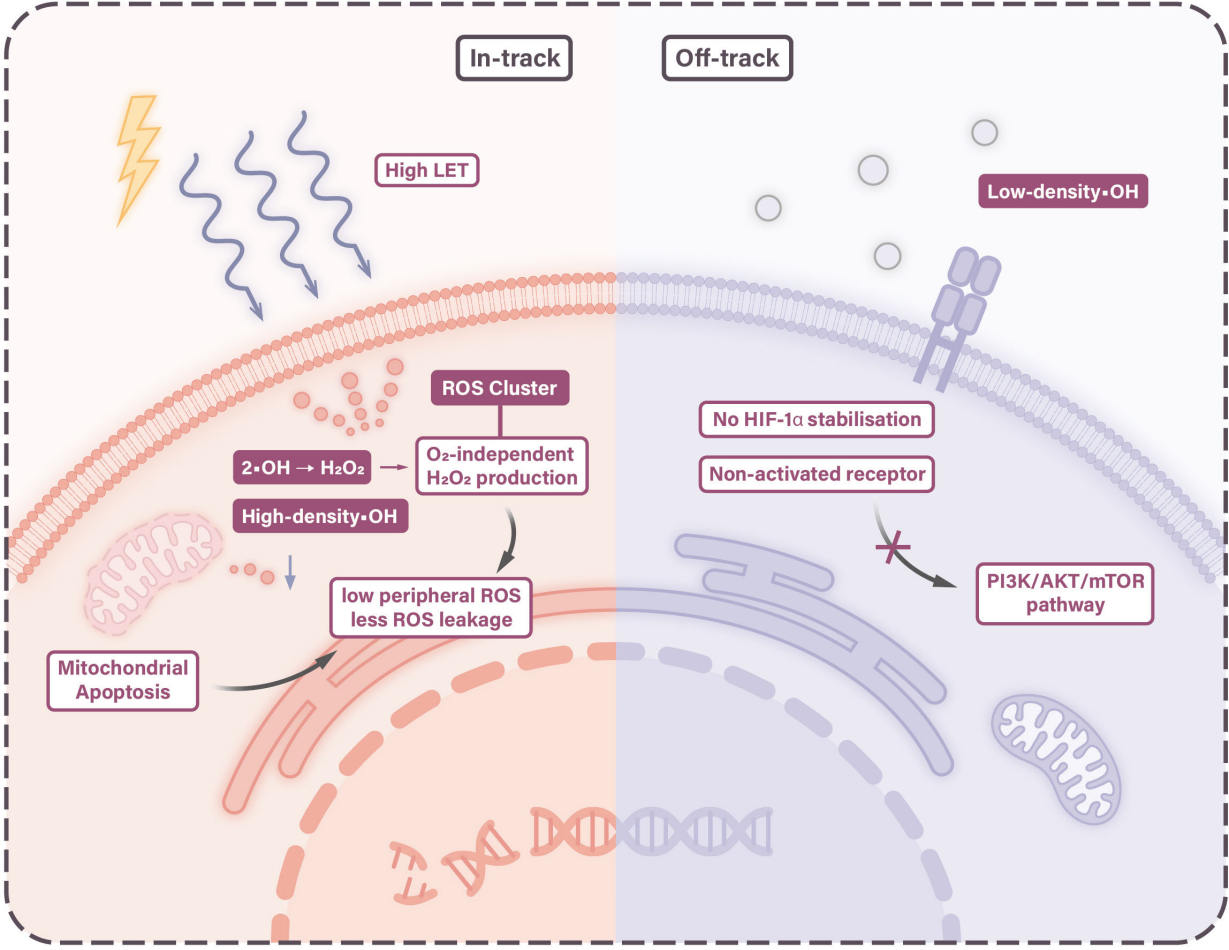
Localized ROS dynamics induced by CIRT. CIRT induces highly localized ROS generation along particle tracks. In-track cells (left) form dense ROS clusters under high-LET conditions, where elevated ·OH concentrations support oxygen-independent H_2_O_2_ production. Although H_2_O_2_ can partially diffuse outward, its concentration remains insufficient to activate pro-survival signaling pathways. Off-track cells (right), the distribution of ROS is sparse and insufficient to mediate HIF-1α stabilization or activate the PI3K/AKT/mTOR pathway. ROS, Reactive Oxygen Species; LET, Linear Energy Transfer; ·OH, hydroxyl radical; H_2_O_2_, hydrogen peroxide; HIF-1α, Hypoxia-inducible factor-1 alpha; PI3K, Phosphatidylinositol 3-kinase; AKT, Protein kinase B; mTOR, mechanistic target of rapamycin.

Multiple studies have indicated that carbon ion irradiation generates lower levels of ROS than photon radiotherapy at equivalent physical doses (76, 77). However, the underlying mechanisms remain incompletely understood. Ken-ichiro et al. reported that, at the same physical dose, carbon-ion irradiation and photon radiotherapy generated comparable total amounts of •OH. Interestingly, with increasing LET, a distinct spatial heterogeneity between high-density and low-density •OH was observed. Specifically, higher-LET carbon ions produced fewer sparsely distributed •OH species that are more prone to diffusion and interaction with the surrounding environment. This finding suggests that •OH generated by high-LET carbon ions is more tightly confined to the track core, whereas fewer ROS escape into the peripheral regions (78). In a human non-small cell lung cancer model, Subtil FSB et al. reported that carbon ion irradiation significantly reduced HIF-1α levels and markedly delayed tumor growth. Since upstream activation of HIF-1α is mediated by ROS, particularly hydroxyl radicals (OH•), these findings suggest that the distinct spatial distribution of ROS induced by carbon ions may differentially modulate intracellular signaling pathways. Similarly, no activation of epidermal growth factor receptor (EGFR) or its downstream signaling pathways was observed following carbon ion irradiation (79, 80) (**Figure 3**).

Up to 80% of non-small cell lung cancer cases exhibit varying degrees of hypoxia, which is a recognized cause of radioresistance (81, 82). In recent years, studies have shown that ROS are not only key mediators of radiation-induced damage but also important effector molecules of innate immune responses and can participate in the activation of multiple immune signaling pathways (83). In this context, the LET characteristics of carbon-ion radiotherapy and its dose distribution, as well as the resulting spatial heterogeneity of ROS generation, may constitute key yet insufficiently understood parameters in regulating tumor immune responses and the development of RILI. Therefore, further studies are needed to systematically elucidate how these physicochemical parameters influence tumor hypoxia, immune regulatory processes, and normal lung tissue toxicity.

## 6 Challenges and future directions

### 6.1 Current limitations

#### 6.1.1 Biological uncertainties in CIRT

Despite its advantages in physical dose distribution and radiobiological effects, substantial uncertainties remain in the accurate characterization and clinical translation of CRT’s biological effects, particularly in normal lung tissue. A central challenge lies in the variability of RBE, which is not a fixed parameter but a complex function influenced by multiple factors, including LET, dose per fraction, total dose, tissue type, and the specific biological endpoint (84). This variability complicates the accurate estimation of biologically effective dose in clinical practice. Currently, RBE-weighted dose distributions are derived from model-based approaches implemented in treatment planning systems; however, different models may yield substantially different RBE estimates due to variations in their underlying assumptions and treatment of LET distributions.

Additional complexity arises from nuclear fragmentation processes. Secondary fragments generated along the beam path exhibit distinct LET spectra and biological effectiveness compared with primary carbon ions. Existing biophysical models differ in how these fragment contributions are incorporated, leading to discrepancies not only in absolute RBE values but also in their spatial distribution, particularly in the distal and fragmentation tail regions. As a result, the biological dose delivered outside the target volume may be subject to greater uncertainty than is reflected in nominal treatment plans (85).

These issues are further amplified in the context of normal lung tissue, where the characterization of RBE remains limited and is largely derived from preclinical data. Most available evidence is based on *in vitro* assays focusing on endpoints such as clonogenic survival, which may not adequately capture complex tissue-level responses, including RP and fibrosis (86). Emerging *in vivo* studies suggest that lung tissue responses to high-LET radiation may differ substantially from those predicted based on photon radiotherapy, particularly with respect to reduced sublethal damage repair and altered fractionation sensitivity. Moreover, RBE estimation is highly sensitive to the assumed α/β ratio, which remains poorly defined for different lung injury endpoints. Variability in α/β assumptions may lead to significant differences in predicted biological dose and, consequently, in risk assessment for normal tissue complications (87).

#### 6.1.2 Thoracic-specific treatment uncertainties: motion, density heterogeneity, range uncertainty, and fragmentation tail

The thoracic region remains one of the most challenging anatomical sites for particle therapy due to substantial respiratory motion, pronounced tissue heterogeneity, and resulting range uncertainties. Respiratory-induced tumor motion, including diaphragm displacement, introduces significant geometric and dosimetric uncertainties. In scanned beam delivery, the temporal interplay between beam delivery dynamics and respiratory motion can lead to dose inhomogeneities within the target (37).

Experimental studies using moving phantoms have demonstrated that even small motion amplitudes can substantially perturb the planned dose distribution in carbon ion therapy, with dose deviations reaching up to 10% in the target center and even larger discrepancies occurring in the penumbra region. Compared with protons, carbon ions appear more sensitive to motion-induced dose distortion, particularly due to their sharper dose gradients and higher dependence on precise range control. In addition to geometric displacement, respiratory motion also induces variations in beam path length, resulting in motion-induced range changes that further compromise dose accuracy (38).

These challenges are compounded by the intrinsic density heterogeneity of lung tissue. The lung is characterized by low density and complex air–tissue interfaces, which may not be fully resolved by conventional CT imaging. As a result, microscopic heterogeneities along the beam path can alter the energy spectrum of the particles, leading to degradation of the Bragg peak and broadening of the distal fall-off. Moreover, respiratory motion dynamically changes lung density, further affecting the WEPL and increasing uncertainty in particle range prediction. These effects may lead to underdosage of the tumor and unintended irradiation of surrounding normal tissues (88–90).

In addition, nuclear interactions between carbon ions and tissue generate lighter secondary fragments that extend beyond the nominal Bragg peak, forming a so-called distal fragmentation tail. This non-negligible dose deposition beyond the target region reduces the sharpness of distal dose fall-off and complicates dose prediction and treatment planning, particularly in the presence of the uncertainties.

### 6.2 Future directions

Considering the physical and biological uncertainties, current research in CIRT for thoracic tumors is increasingly directed toward both technological refinement and translational investigation. Continued advances in motion management, image guidance, and adaptive treatment strategies—supported by four-dimensional imaging, rescanning techniques, and increasingly sophisticated planning frameworks—are likely to improve the robustness and precision of dose delivery in the dynamic thoracic environment. In parallel, emerging approaches such as artificial intelligence–assisted planning and adaptive workflows may further enhance the ability to account for inter- and intra-fractional variability.

Beyond technical refinement, there is a growing need for high-quality clinical evidence to define the role of CIRT in thoracic oncology. Prospective clinical trials and multi-institutional collaborations will be essential to validate its therapeutic efficacy, refine its safety profile, and optimize patient selection. In particular, randomized comparisons with photon and proton therapies, as well as the establishment of international registries, may help overcome current limitations related to small sample sizes and heterogeneity, while identifying patient subgroups that are most likely to benefit from CIRT.

From a biological and translational perspective, increasing attention is being directed toward the integration of CIRT with emerging therapeutic strategies. The unique radiobiological properties of carbon ions, including high-LET–induced complex DNA damage and immunogenic cell death, provide a strong rationale for combination with immunotherapy. Early evidence suggests that such combinations may enhance anti-tumor immunity while maintaining an acceptable safety profile, highlighting a promising avenue for improving both tumor control and normal tissue protection.

In addition, the potential of CIRT to mitigate RILI warrants further systematic investigation. Elucidating the underlying cellular and molecular mechanisms—such as modulation of inflammatory pathways, ROS distribution, and exosome- or miRNA-mediated signaling—may provide important insights into its protective effects. Integrating these mechanistic findings with clinical and dosimetric data, including the identification of predictive biomarkers (e.g., cytokine profiles and imaging-based parameters), could enable more accurate risk stratification and support personalized treatment strategies.

Finally, emerging modalities such as ultra-high dose rate (FLASH) irradiation may further expand the therapeutic window of CIRT by reducing normal tissue toxicity (91). Together with ongoing advances in delivery systems, imaging technologies, and biological modeling, these developments point toward a more integrated and individualized treatment paradigm, in which physical precision, biological effectiveness, and patient-specific factors are jointly optimized.

## Glossary

3D-CRT: Three-Dimensional Conformal Radiotherapy
AKT: AKT Kinase (Protein Kinase B)
CIRT: Carbon Ion Radiotherapy
DAMPs: Damage-Associated Molecular Patterns
DSBs: Double-Strand Breaks
DVH: Dose–Volume Histogram
ECM: Extracellular Matrix
EGFR: Epidermal Growth Factor Receptor
H₂O₂: Hydrogen Peroxide
high-LET: High Linear Energy Transfer
HMGB1: High-Mobility Group Box 1
IFN-γ: Interferon-Gamma
IL-1: Interleukin-1
IL-4: Interleukin-4
IL-6: Interleukin-6
IL-10: Interleukin-10
IL-12: Interleukin-12
IL-13: Interleukin-13
IMRT: Intensity-Modulated Radiotherapy
IP: Interstitial Pneumonia
LET: Linear Energy Transfer
LC: Local Control
LC-IP: Lung Cancer with Interstitial Pneumonia
MLD: Mean Lung Dose
NHEJ: Non-Homologous End Joining
NSCLC: Non-Small Cell Lung Cancer
O₂⁻: Superoxide Anion
OS: Overall Survival
RBE: Relative Biological Effectiveness
RILI: Radiation-Induced Lung Injury
ROS: Reactive Oxygen Species
RP: Radiation Pneumonitis
RPF: Radiation Pulmonary Fibrosis
SSBs: Single-Strand Breaks
TGF-β: Transforming Growth Factor-Beta
TNF-α: Tumor Necrosis Factor-Alpha
VMAT: Volumetric Modulated Arc Therapy
